# Dataset on bus mobility and environmental indicators from Rio de Janeiro

**DOI:** 10.1038/s41597-025-05755-6

**Published:** 2025-09-26

**Authors:** Diego Carvalho, Vinícius Vancellote, Pablo Moreira Casais, João Luiz Carabetta, Bruno Almeida, Fabio Porto, Eduardo Mendes, Renato Rocha Souza, Douglas de O. Cardoso, Peter Fernandes Wanke, Rafael Barbastefano, Rafaelli Coutinho, Diego Brandão, Miguel Diaz-Cacho, André Mendes, Eduardo Ogasawara

**Affiliations:** 1 Federal Centre for Technological Education of Rio de Janeiro (CEFET/RJ), Rio de Janeiro, Brazil; 2 Prefeitura do Rio de Janeiro, Rio de Janeiro, Brazil; 3 National Laboratory of Scientific Computing, Petrópolis, Brazil; 4 FGV, São Paulo, Brazil; 5 FGV, Rio de Janeiro, Brazil; 6 Faculty of Economics, University of Coimbra, Coimbra, Portugal; 7 EBAPE - Brazilian School of Public and Business Administration, Getulio Vargas Foundation, Rio de Janeiro, Brazil; 8 Department of Systems Engineering and Automatics, Universidade de Vigo, Vigo, Spain; 9 VALORIZA, Research Centre for Endogenous Resource Valorization, Polytechnic Institute of Portalegre, Campus Politécnico, Portalegre, Portugal

**Keywords:** Engineering, Mathematics and computing

## Abstract

The quality of public transport is essential when considering urban mobility in large cities. Several factors, such as the increase in urban population, rain, and traffic events, can impact mobility, causing congestion. Addressing this issue is essential for the population and is part of the UN’s 2030 Agenda for Sustainable Development goals. Integrating data from different sources is crucial to understanding and planning urban traffic. This work aims to provide a dataset with spatiotemporal information on the mobility of municipal buses, including the estimated emission of polluting gases and the rainfall volume in Rio de Janeiro from 2014 to 2023. Its format facilitates integration with other Rio de Janeiro City Hall datasets, enabling the increase and deepening of the analyses. This work is the first to combine data from bus observation with positional information on neighborhoods and rainfall regions, rainfall volumes, and pollutant gas emissions. Thus, its availability opens opportunities for research topics involving public transport associated with environmental indicators and data science with time series studies and positional data.

## Background & Summary

In 1950, approximately 30% of the global population resided in urban areas. This percentage has now surpassed half and is projected to reach 80% by 2050^1^. Citizens have been relocating to cities due to convenient access to other individuals, employment, and opportunities. An indirect consequence of this migration is the occurrence of traffic jam, which affects people’s accessibility^2^. Therefore, public transportation plays a crucial role in the population’s daily lives as it is a critical infrastructure for the economy^3^.

Today, the motorized transportation sector is responsible for emitting over 15% of the world’s polluting gases. Consequently, reducing vehicular traffic in cities has also become imperative to mitigate the impacts of climate emergencies^4,5^. This objective is part of the goals outlined in the 2030 Agenda for Sustainable Development developed by the United Nations (UN).

More broadly, understanding urban mobility is essential for transportation planning and other urban processes, such as the spread of epidemics^4^. The recent surge in urban data volumes has paved the way for the emergence of a new field of study known as the *Science of Cities*. However, developing a simple model to explain the dominant mechanisms governing the formation and evolution of mobility patterns remains elusive^6^.

Since traffic jam vary across different times and locations, incorporating spatiotemporal data enhances the reliability of assessing their impacts^2^. Thus, this work aims to provide a dataset called Carioca_MapBus that contains more than just spatiotemporal information on the mobility of municipal buses in Rio de Janeiro. It integrates data from multiple sources, expanding research on various subjects related to the study of mobility. For instance, this information includes estimates of pollutant gases emissions such as Carbon Monoxide (*CO*), Carbon Dioxide (*CO*_2_), Hydrocarbons (*HC*), and Nitrogen Oxides (*NO*_*x*_), as well as historical records of daily rainfall volume, administrative regions, bus garages, public transport terminals, and areas of interest (e.g., express corridors).

The availability of the Carioca_MapBus dataset presents opportunities for researching urban mobility in a megalopolis and its connection to environmental issues. It also provides an opportunity to conduct data science studies involving space-time series. Examples of these opportunities are presented in this paper.

## Methods

### Buses data sources

The dataset stems from ongoing data collection efforts initiated in 2014 by the CEFET’s Laboratory of Computational Intelligence in Engineering and Management (LINCE), coinciding with the SIURB (Municipal Urban Information System) implementation by the City Hall of Rio de Janeiro. This system began providing information related to the public transit system of the city of Rio de Janeiro^7^. Each vehicle in the Rio bus system contains an embedded board programmed to transmit trip information such as vehicle ID, geopositioning, service line, and instant velocity at regular intervals. The City Hall collects this trip information and offers a web access point containing data about every vehicle in the system.

The information on the City Hall’s access point resembles an aerial photograph of the city, providing the most up-to-date data for every bus. However, fetching data at timed intervals may lead to duplicated information if a bus still needs to update its data between fetches. Besides, the service does not provide historical data.

### Dataset preparation

**Fig. 1:**
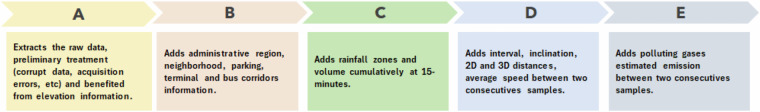
The pipeline for the Data Summary Table building for the Carioca_MapBus. Each stage augments the usefulness of the dataset, adding different domain information for every observation throughout the process. Stage A assures the quality of the data and adds the elevation coordinate. The next stage attaches the administrative information conveyed to the positioning associated with each observation. Stage C assimilates the rainfall-related information. Stage D adds variables associated with the vehicle displacement. Stage E computes polluting emissions related to the observation.

The dataset was created in five stages. Each stage includes metadata and new variables to make the information more useful. The pipeline of stages is illustrated in Fig. 1, and a Data Summary Table (DST) file results from each stage, providing descriptive statistics, provenance, and versioning. Stage (A) extracts and treats the raw data to fix errors and adds information about topographic elevation. Stage (B) adds data about the administrative regions of Rio de Janeiro city, neighborhoods, garages, and bus terminals. Then, Stage (C) inserts the rainfall zones and volumes. Stage (D) includes sample intervals, average speeds, elevations, and bus travel distances. Finally, Stage (E) incorporates estimates of polluting gas emissions. Each stage is detailed as follows.

**Fig. 2:**
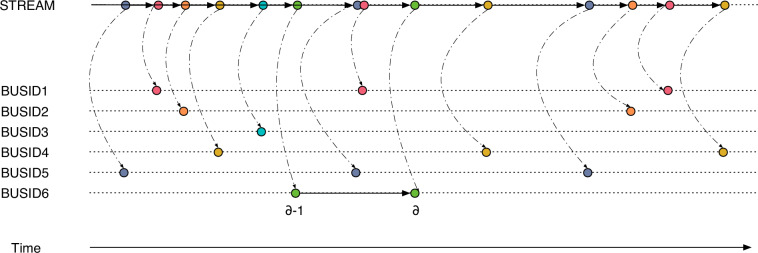
The data stream is composed of unique observations that indicate a change in the transit system.

**Stage (A)** extracts the raw data from the Rio de Janeiro City Hall monitoring facility (Data.Rio: https://www.data.rio/) and stores it in our servers. The raw data consists of observations on buses operating in Rio de Janeiro, collected into a file every minute from 04/16/2014 to 06/30/2023. Each entry, depicted in Table 1, displays the most recent data collected for each vehicle, including the time captured from an embedded GPS. This information includes the date, hour, minute, and second (GPSTIMESTAMP, or *t*), a unique identification number for the bus (BUSID), the service line on which it was operating at the time of observation (LINE), the latitude (LATITUDE), longitude (LONGITUDE), and the instant speed recorded through a GPS sensor installed on the vehicle (VELOCITY). Then, the processing progresses by grouping each day’s raw data (1,440 files per day) and removing duplicated data, transmission errors, and gibberish data, such as vehicles with negative instant speeds or above 120 km/h. Finally, we clip any observation outside a polygon defined by the municipality border (The bounding box comprises: maximum latitude −22.6^°^, minimum latitude −23.2^°^, maximum longitude −43.0^°^, minimum longitude −44.0^°^). and map the remaining observations with the elevation (ELEVATION, integrated with data from the Shuttle Radar Topography Mission^8^). We process the result as a data stream, as shown at the top of Fig. 2. This stream comprises unique observations, each indicating a change in the state of the transit system. The following (B and C) stages tag each observation with position-related information. Then, we split this stream into several streams in stage D: one stream per vehicle, suitable for displacement-related calculations.

**Table 1:** Raw data from the Data.Rio datasets.

Data Type	Attribute	Description
timestamp	GPSTIMESTAMP	GMT-0 Date
string	BUSID	Bus serial number
string	LINE	Bus line
double	LATITUDE	Latitude
double	LONGITUDE	Longitude
double	VELOCITY	Instant speed measured by GPS

**Fig. 3:**
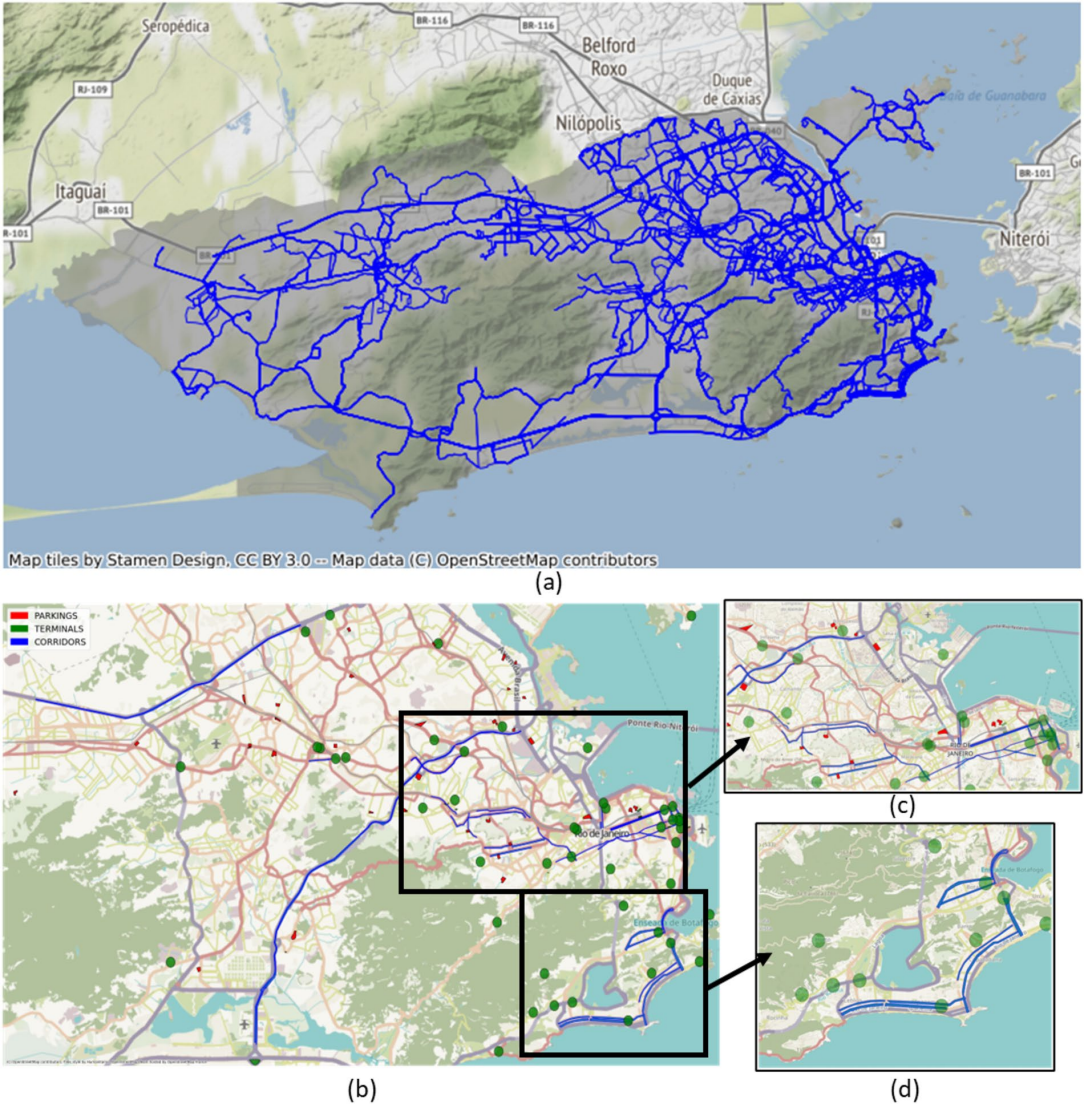
Rio de Janeiro’s bus public transit system: (**a**) Metropolitan service routes network; (**b**) Parking garages in red, bus terminals in green, and express corridors in blue; (**c**) North zone zoom; and (**d**) South zone zoom.

**Stage (B)** tags administrative labels for each observation. The first group of labels comprises the regional subdivision of the municipality. It indicates the administrative region (ADMINISTRATIVEREGION) and the neighborhood (NEIGHBORHOOD). The tagging process uses the observation’s geopositioning (latitude and longitude) and GeoJSON files, representing the regions provided by the Rio de Janeiro City Hall (The dataset with the delimitation of neighborhoods is available^9^). Table 2 presents the city’s division by administrative regions and neighborhoods, respectively. Additionally, we also provide transit-related information, indicating if an observation lies on a parking lot (PARKING) or a bus terminal (TERMINAL). For the parking GeoJSON file, clustering techniques are used to group observations with zero speed at night and in the early morning hours, with manual inspection of satellite imagery. We use a similar process to create a terminal GeoJSON file. Figure 3(a) depicts Rio de Janeiro’s bus public transit system with each Metropolitan service route, and Fig. 3(b) shows the parking garages in red, bus terminals in green, and express corridors in blue.

**Table 2:** Administratives Regions and Neighborhoods.

ADM REGION	ADM REGION NAME	NEIGHBORHOOD	NEIGHBORHOODS NAMES (ORDERED)
1	PORTUARIA	002, 003, 001, 004	Gamboa, Santo Cristo, Saúde, Caju
2	CENTRO	005, 161	Centro, Lapa
3	RIO COMPRIDO	008, 007, 009, 006	Cidade Nova, Rio Comprido, Estácio, Catumbi
4	BOTAFOGO	016, 018, 015, 017, 019, 020, 022, 021	Glória, Catete, Flamengo, Laranjeiras, Cosme Velho, Botafogo, Urca, Humaitá
5	COPACABANA	024, 023	Copacabana, Leme
6	LAGOA	028, 027, 029, 026, 025, 031, 030	Jardim Botânico, Lagoa, Gávea, Leblon, Ipanema, São Conrado, Vidigal
7	SAO CRISTOVAO	012, 010, 158, 011	Benfica, São Cristóvão, Vasco da Gama, Mangueira
8	TIJUCA	032, 033, 034	Praça da Bandeira, Tijuca, Alto da Boa Vista
9	VILA ISABEL	036, 035, 038, 037	Vila Isabel, Maracanã, Grajaú, Andaraí
10	RAMOS	042, 041, 040, 039	Olaria, Ramos, Bonsucesso, Manguinhos
11	PENHA	044, 045, 043	Penha Circular, Brás de Pina, Penha
12	INHAUMA	055, 054, 056, 050, 053, 052	Engenho da Rainha, Inhaúma, Tomás Coelho, Higienópolis, Del Castilho, Maria da Graça
13	MEIER	071, 069, 070, 065, 066, 064, 051, 068, 058, 063, 060, 059, 057, 061, 067, 062	Pilares, Piedade, Abolição, Cachambi, Engenho de Dentro, Todos os Santos, Jacaré, Encantado, Rocha, Méier, Sampaio, Riachuelo, São Francisco Xavier, Engenho Novo, Água Santa, Lins de Vasconcelos
14	IRAJA	076, 075, 077, 074, 072, 073	Irajá, Vista Alegre, Colégio, Vila da Penha, Vila Kosmos, Vicente de Carvalho
15	MADUREIRA	087, 086, 090, 084, 089, 085, 083, 088, 080, 081, 082, 079, 078	Honório Gurgel, Rocha Miranda, Marechal Hermes, Vaz Lobo, Bento Ribeiro, Turiaçú, Madureira, Osvaldo Cruz, Cavalcanti, Engenheiro Leal, Cascadura, Quintino Bocaiúva, Campinho
16	JACAREPAGUA	125, 124, 123, 122, 120, 121, 119, 116, 115, 117	Vila Valqueire, Praça Seca, Tanque, Taquara, Freguesia (Jacarepaguá), Pechincha, Curicica, Anil, Jacarepaguá, Gardênia Azul
17	BANGU	140, 142, 141, 160, 162, 163	Padre Miguel, Senador Camará, Bangu, Gericinó, Vila Kennedy, Jabour
18	CAMPO GRANDE	143, 145, 147, 146, 144	Santíssimo, Senador Vasconcelos, Cosmos, Inhoaíba, Campo Grande
19	SANTA CRUZ	148, 149, 150	Paciência, Santa Cruz, Sepetiba
20	ILHA DO GOVERNADOR	098, 097, 104, 101, 103, 102, 096, 100, 099, 095, 093, 094, 092, 091, 105	Freguesia (Ilha), Bancários, Galeão, Tauá, Portuguesa, Moneró, Cocotá, Jardim Carioca, Jardim Guanabara, Praia da Bandeira, Cacuia, Pitangueiras, Zumbi, Ribeira, Cidade Universitária
21	PAQUETA	013	Paquetá
22	ANCHIETA	107, 106, 108, 109	Anchieta, Guadalupe, Parque Anchieta, Ricardo de Albuquerque
23	SANTA TEREZA	014	Santa Teresa
24	BARRA DA TIJUCA	131, 129, 130, 127, 128, 132, 126, 133	Vargem Grande, Camorim, Vargem Pequena, Itanhangá, Barra da Tijuca, Recreio dos Bandeirantes, Joá, Grumari
25	PAVUNA	114, 159, 111, 113, 110, 112	Pavuna, Parque Colúmbia, Acari, Costa Barros, Coelho Neto, Barros Filho
26	GUARATIBA	151, 153, 152, 164	Guaratiba, Pedra de Guaratiba, Barra de Guaratiba, Ilha de Guaratiba
27	ROCINHA	154	Rocinha
28	JACAREZINHO	155	Jacarezinho
29	COMPLEXO DO ALEMÃO	156	Complexo do Alemão
30	COMPLEXO DA MARE	157	Maré
31	VIGARIO GERAL	048, 049, 046, 047	Vigário Geral, Jardim América, Cordovil, Parada de Lucas
33	REALENGO	135, 139, 138, 136, 137, 134	Vila Militar, Realengo, Magalhães Bastos, Campo dos Afonsos, Jardim Sulacap, Deodoro
34	CIDADE DE DEUS	118	Cidade de Deus

**Fig. 4:**
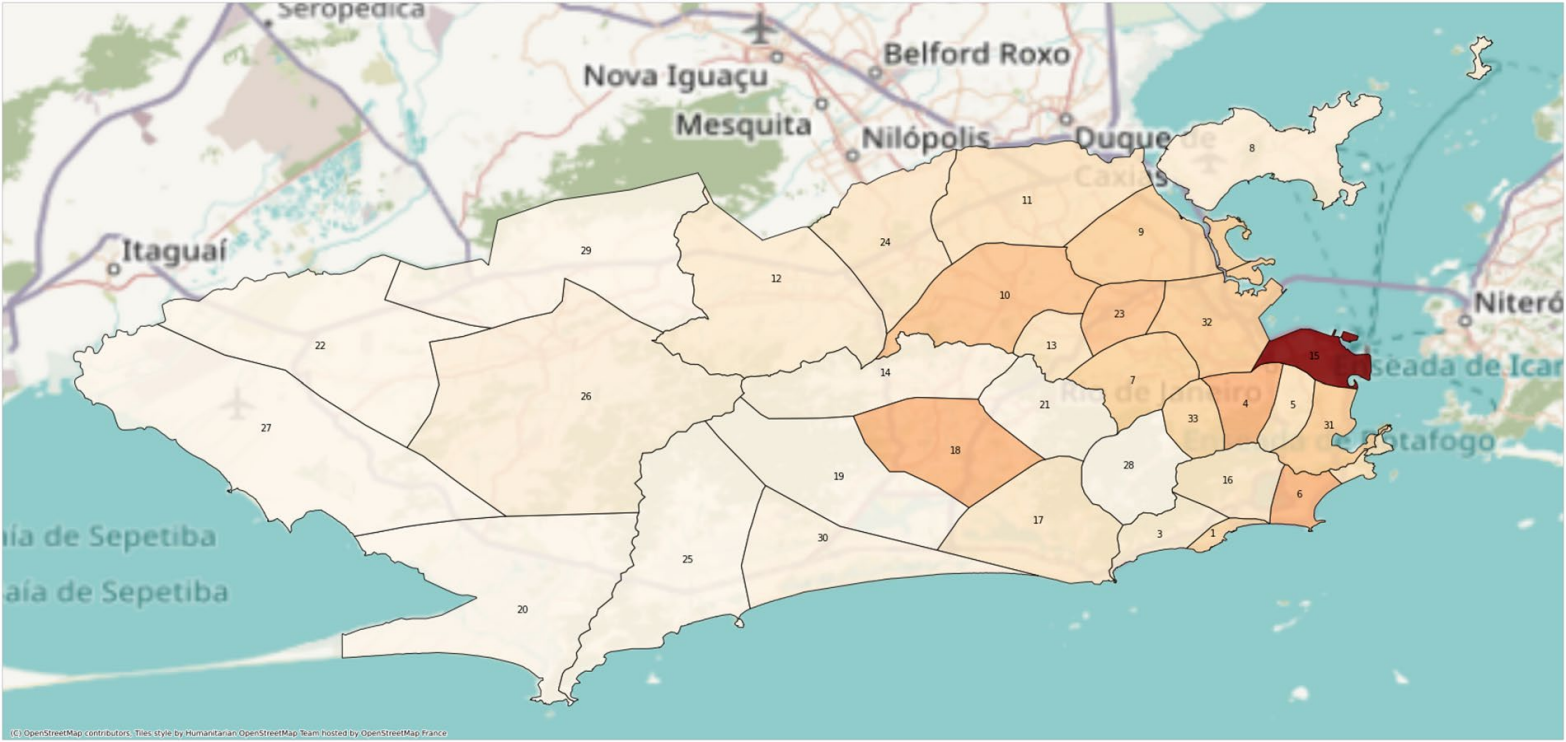
The City Hall of Rio de Janeiro divided the city into Rainfall Zones, where pluviometric measurement sensors are installed and monitored. The figure shows each Rainfall Zone, and the color varying from white to red indicates the number of observations in the database per area unit (density). The Table 7. provides the name for each zone.

**Stage (C)** includes information about rainfall volume (RAINFALLVOLUME) and zone (RAINFALLZONE), shown in Fig. 4. Rainfall volumes are available cumulatively at 15-minute intervals from 2014 to 2023 in the Alerta Rio system (The rainfall volumes dataset is available^9^). Thus, for each observation, we first identify the zone and then the rainfall volume associated with the 15-minute interval of its occurrence.

**Stage (D)** splits the data stream into sections, one for each vehicle, as shown in Fig. 2. For every observation sequence *δ*, we compute variables related to the previous observation (*δ* − 1). Each value is recorded in an attribute linked with the observation sequence *δ*. The variables include the two-dimensional distance *hd*_*δ*_ (the haversine distance between observations sequences *δ* − 1 and *δ*), the height *h*_*δ*_ determined using the elevations (*e*_*δ*_ and *e*_*δ*−1_), the interval *i*_*δ*_, the three-dimensional distance *d*_*δ*_, and the average speed *s*_*δ*_. Table 3 summarizes the notation used.

**Table 3:** Description of used notations.

Notation	Description	Attributes
*δ*	Sequence of the observation	ID
*t*_*δ*_	Recording timestamp of observation *δ*	GPSTIMESTAMP
*e*_*δ*_	Elevation via spatial sensing of observation *δ*	ELEVATION
*dh*_*δ*_	Two-dimensional distance between observations *δ* and *δ* − 1	HAVERSINEDISTANCE
*h*_*δ*_	Height difference between elevations *e*_*δ*_ and *e*_*δ*−1_	HEIGHT
*i*_*δ*_	Time difference between observations *δ* and *δ* − 1	INTERVAL
*d*_*δ*_	Three-dimensional distance between observations *δ* and *δ* − 1	DISTANCE
*s*_*δ*_	Average speed between observations *δ* and *δ* − 1	SPEED
*a*_*δ*_	Acceleration or deceleration of the bus associated with observation *δ*	n/a
*VSP*_*δ*_	Vehicle specific power used to determine average emission rates of CO, CO_2_, NO_*x*_, and HC of the bus associated with observation *δ*	CO, CO2, NOx, HC

Equation (1) corresponds to the two-dimensional distance *hd*_*δ*_ traveled between observations sequences *δ* and *δ* − 1, computed with the Haversine formula (i.e. distance between the two points along a great circle of the sphere), where *r* is the radius of the Earth, *θ*_*δ*−1_ and *θ*_*δ*_ are the latitudes, and *λ*_*δ*−1_ and *λ*_*δ*_ are the longitudes of each observation.

1 $$h{d}_{\delta }=2r\arcsin \left(\sqrt{si{n}^{2}\left(\frac{{\theta }_{\delta }-{\theta }_{\delta -1}}{2}\right)+\cos ({\theta }_{\delta -1})\cos ({\theta }_{\delta }){\sin }^{2}\left(\frac{{\lambda }_{\delta }-{\lambda }_{\delta -1}}{2}\right)}\right)$$

Let *h*_*δ*_ be the height difference between two subsequent observations of the same bus, as described in Equation (2).

2 $${h}_{\delta }={e}_{\delta }-{e}_{\delta -1}$$

The interval *i*_*δ*_ refers to the time difference between a particular vehicle’s current and previous observation. It is computed as follows: if the interval is less than or equal to 240 seconds, Equation (3) is applied. On the other hand, if the computed interval is more than 240 seconds, it is assumed there was a failure in the communication system. The resulting value is set to NA (not available) (Equation (4)).

3 $${i}_{\delta }={t}_{\delta }-{t}_{\delta -1},{i}_{\delta }\le 240s$$

4 $${i}_{\delta }={\mathtt{NA}},{i}_{\delta } > 240s$$

Let *d*_*δ*_ be the three-dimensional distance traveled between two observations considering the two-dimensional distance and height. It is computed from the Pythagorean Theorem using the information *hd*_*δ*_ and *h*_*δ*_, as described in Equation (5).

5 $${d}_{\delta }=\sqrt{h{d}_{\delta }^{2}+{h}_{\delta }^{2}}$$

The average speed between two observations, *s*_*δ*_, is obtained by dividing the three-dimensional distance (*d*_*δ*_) by the interval (*i*_*δ*_), according to Equation (6).

6 $${s}_{\delta }=\frac{{d}_{\delta }}{{i}_{\delta }}$$

**Stage (E)** finally inserts the information about the estimated emission of polluting gases *CO*, *CO*_2_, *HC*, and *NO*_*x*_ into the dataset. We adopt a bottom-up approach to compute these emissions estimates, which is valuable for identifying emission sources and, thus, essential for developing public policies and implementing mitigation measures. However, it should be noted that the methodology employed lacks a comprehensive analysis of emission sources, considering the nature and type of activity responsible for emissions. In this work, we only use buses circulating in Rio de Janeiro city as a source of emissions. Thus, we use the Vehicle Specific Power (VSP) model to estimate modal emission rates and associate them with average emission rates for diesel transport buses^10^. The VSP model defines the power per unit mass of the source (kW/ton), making it a convenient way to estimate a vehicle’s emissions and using several factors, such as vehicle acceleration, road slope, tire rolling resistance, and aerodynamic resistance, influence absolute power^11^. A limitation of this estimate is that the vehicle’s weight affects the emissions of heavy-duty diesel vehicles^12^, and the passenger counting in the element fleet is unavailable. The VSP is computed based on the bus speed *s*_*δ*_, the acceleration or deceleration of the vehicle *a*, and the slope of the road $\sin (\alpha )={h}_{\delta }/{d}_{\delta }$. We first compute the acceleration *a* using Equation (7), such that *s*_*δ*_ − *s*_*δ*−1_ is the speed variation, i.e., the difference between the current observation speed and the previous one, and the interval *i*_*δ*_, i. e., the difference between the current observation time and the previous one,

7 $${a}_{\delta }=\frac{{s}_{\delta }-{s}_{\delta -1}}{{i}_{\delta }}.$$

For two successive observations, the *VSP*_*δ*_ is computed using Equation (8). Then, the average modal emission rates for *CO*_2_, *CO*, *NO*_*x*_, and *HC* are obtained using Table 4.

8 $$VS{P}_{\delta }={s}_{\delta }\left[\left(9.81{a}_{\delta }\frac{{h}_{\delta }}{{d}_{\delta }}+0.092\right)+0.00021{s}_{\delta }^{2}\right]$$

The dataset provides the computed estimates in mass per second, and it is still necessary to multiply these emission rates by the interval between the two observations to obtain the total mass of pollutants emitted. Finally, we add the emissions obtained for the entire bus line journey to bring the total emissions of *CO*, *CO*_2_, *HC*, and *NO*_*x*_.

**Table 4:** Average pollutant emission rates from diesel buses^10^.

VSP range	VSP mode	Average modal emission rates			
		CO_2_ (g/s)	CO (g/s)	NO_*x*_ (g/s)	HC (mg/s)
*VSP* ≤ 0	1	2.4	0.009	0.04	1.23
0 < *VSP* < 2	2	7.8	0.036	0.13	1.7
2 ≤ *VSP* < 4	3	12.5	0.045	0.18	1.75
4 ≤ *VSP* < 6	4	17.1	0.072	0.22	1.84
6 ≤ *VSP* < 8	5	21.2	0.085	0.24	1.94
8 ≤ *VSP* < 10	6	24.8	0.091	0.26	2.05
10 ≤ *VSP* < 13	7	27.6	0.084	0.28	2.08
*VSP* ≥ 13	8	29.5	0.062	0.31	2.15

### Data gaps

The path between data generation in the vehicle and the final storage is not error-prone and presents points of failure. Although we developed an extensive dataset, it is only comprehensive for some periods.

## Data Records

The dataset Carioca_MapBus is publicly available in OSF^13^. Table 5 shows the available dataset and related files for download. The Open Science Framework (OSF) repository, within the research project Carioca_MapBus^13^, makes available all necessary links to access raw and processed data, file descriptions, GTFS, and external links^9^, including, among others, population data.

**Table 5:** Overview of data files/datasets.

Label	File type (file extension)	Data repository and identifier
data	Folder	OSF: https://osf.io/yptkj
dst-retrieval.py	Python	OSF: https://osf.io/jzyfn
readme	PDF	OSF: https://osf.io/6h3wy
config	CSV	OSF: https://osf.io/zp9ds
raw data	Folder/parquet	OSF: https://osf.io/yptkj/wiki/ImportantLinks/

## Technical Validation

To ensure the dataset’s reliability, we critically assess three factors: the quality of the information source, discontinuities, and various aspects of the data, as outlined below.

The dataset consists of 3,228 files structured as data summary tables (DST) in Parquet format^14^, comprising over 9 billion observations across 25 attributes detailed in Table 6. These files are categorized into five types of DST files representing different themes: positioning information (DST-A), city administrative data (DST-B), rainfall data (DST-C), displacement information (DST-D), and emissions (DST-E). One attribute, shared across all DST themes, uniquely identifies observations and correlates DSTs. At DST-A, the attribute ID is a primary key, whereas at DST-B, DST-C, DST-D, and DST-E, the attribute ID is both a primary key and a foreign key to DST-A. In class A, two attributes identify the vehicle and the service line within the Rio de Janeiro City Hall, facilitating the identification of observed buses and monitoring the consistency of their routes with operational lines.

**Table 6:** Description of DSTs.

DST-A		
Data Type	Attribute	Description
index	ID	Primary key
timestamp	GPSTIMESTAMP	GMT-0 Date
string	BUSID	Bus serial number
string	LINE	Bus line
double	LATITUDE	Latitude
double	LONGITUDE	Longitude
double	VELOCITY	Instant velocity measured by the embedded GPS (m/s)
double	ELEVATION	Position’s elevation extracted from SRTM (Shuttle Radar Topography Mission) data^8^
string	SWVERSION	Pipeline version

DST-B		
index	ID	Foreign key (same DST-A ID)
string	ADMINISTRATIVEREGION	Administrative region ID
string	NEIGHBORHOOD	Neighborhood ID
double	PARKING	Bus parking lot ID
double	TERMINAL	Bus terminal ID
double	CORRIDOR	Express bus corridor ID
string	SWVERSION	Pipeline version

DST-C		
index	ID	Foreign key (same DST-A ID)
string	RAINFALLZONE	Rainfall Zone ID
string	RAINFALLVOLUME	Rainfall volume over the last 15 minutes
string	SWVERSION	Pipeline version

DST-D		
index	ID	Foreign key (same DST-A ID)
double	HAVERSINEDISTANCE	Haversine distance between two observations (m)
double	HEIGHT	SRMT1 height between two observations
timestamp	INTERVAL	Time between two observations (s)
double	DISTANCE	Three-dimensional distance between two observations (m)
double	SPEED	Average speed between two observations (m/s)
string	SWVERSION	Pipeline version

DST-E		
index	ID	Foreign key (same DST-A ID)
double	CO	Estimation of the mass of carbon monoxide emitted between two observations (g/s)
double	CO2	Estimation of the mass of carbon dioxide emitted between two observations (g/s)
double	NOx	Estimation of the mass of nitrogen monoxide emitted between two observations (g/s)
double	HC	Estimation of the mass of hydrocarbon emitted between two observations (g/s)
string	SWVERSION	Pipeline version

Attributes are computed based on combinations of time, space, and metrics related to the environment and mobility. Temporal attributes include the bus’s GPS timestamp for tracking observations over time, while space attributes encompass latitude, longitude, elevation, administrative region, neighborhood, parking, terminal, corridor, and rainfall zone. One attribute represents the rainfall volume in the last 15 minutes, and six attributes are associated with bus mobility metrics, including 2D and 3D distances, elevation, interval, and instant and average speeds. Finally, four attributes estimate gas emissions for CO, CO_2_, HC, and NO_*x*_. Note that all buses in Rio de Janeiro use a diesel-based fuel called ARLA 32 (Automotive Liquid Reducing Agent).

The dataset contains one DST file for each day from April 16, 2014, to June 30, 2023, comprising 3,362 files. In this period, 134 days have no information due to acquisition issues, representing less than 4% of the period. The lack of information is related to different reasons, such as (i) instability in the City Hall server that made the data available, (ii) instability in the server that collected the data, (iii) temporary interruption in the availability of data due to the occurrence of some maintenance, and (iv) data corruption during generation on the server.

The City Hall of Rio de Janeiro divides the city into Rainfall Zones, deploying and monitoring pluviometric measurement sensors. In Fig. 4, Rainfall Zones are displayed, showing the density of observations per area unit. Neighborhoods near Guanabara Bay, associated with the Center Zone of the city, exhibit more observations than those in the West Zone, reflecting the daily commuting pattern where people travel from residential zones to downtown for work.

**Fig. 5:**
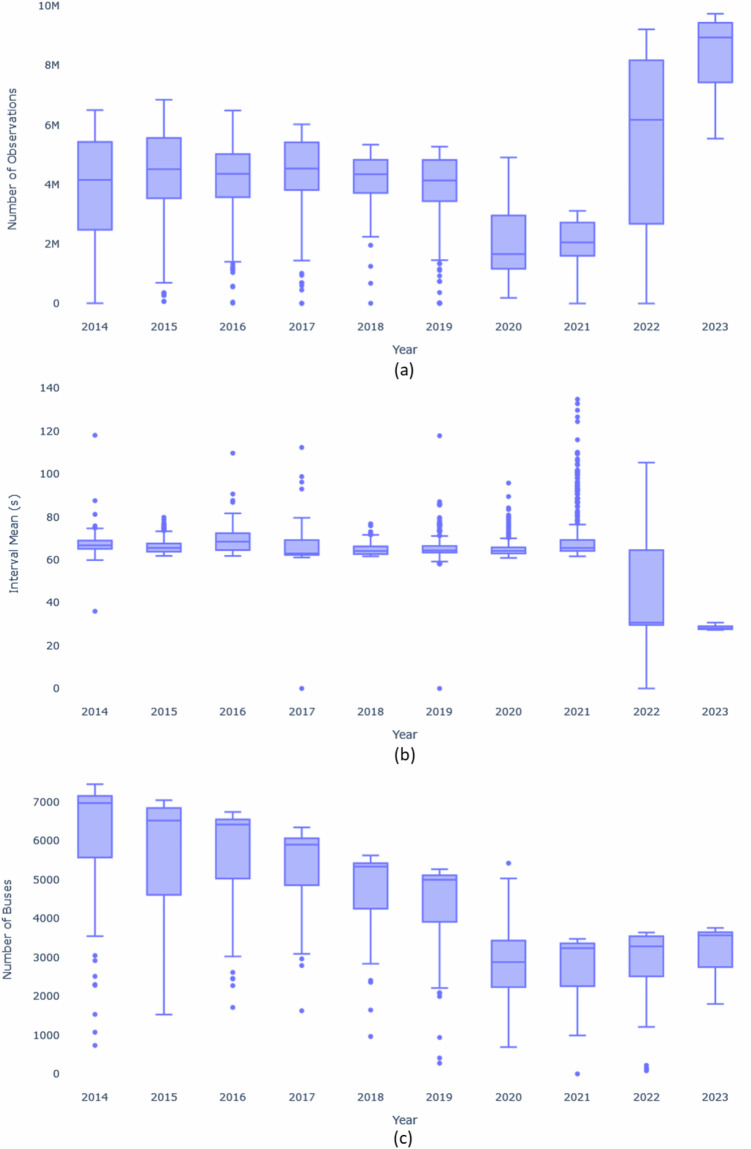
The box plot of the average number of distinct observations per day in the database (**a**), the average interval time between observations in seconds on each day in the database (**b**), and the average number of vehicles per day in the database (**c**).

**Fig. 6:**
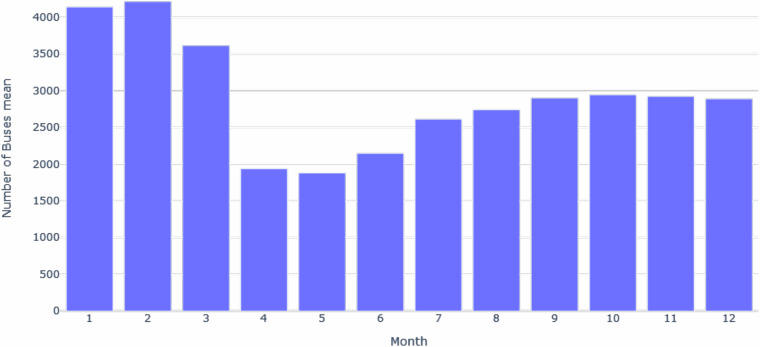
The horizontal axis shows 2020’s months, and the vertical axis shows the mean of buses circulating every day.

Figure 5(a) illustrates the average daily observations for each year. There was a peak in 2015, with the average surpassing 4 million daily observations. However, this number drastically reduced to around 2 million by 2020, likely due to the COVID-19 pandemic. Figure 5(b) shows the average time interval between observations per year, with dispersion increasing in 2020 due to the pandemic and decreasing in 2023 due to City Hall improvements. Figure 5(c) displays the distribution of the average number of daily buses in circulation per year, showing a decline over the years, likely correlating with the decrease in observations. A notable decrease in this indicator occurred in 2020, coinciding with the onset of COVID-19 spread in Rio de Janeiro, as depicted by the lower limit of the boxplot. Additionally, Figure 6 illustrates the decline in the number of buses in circulation starting from March 2020, coinciding with the reinforcement of lockdown measures in Brazil.

**Fig. 7:**
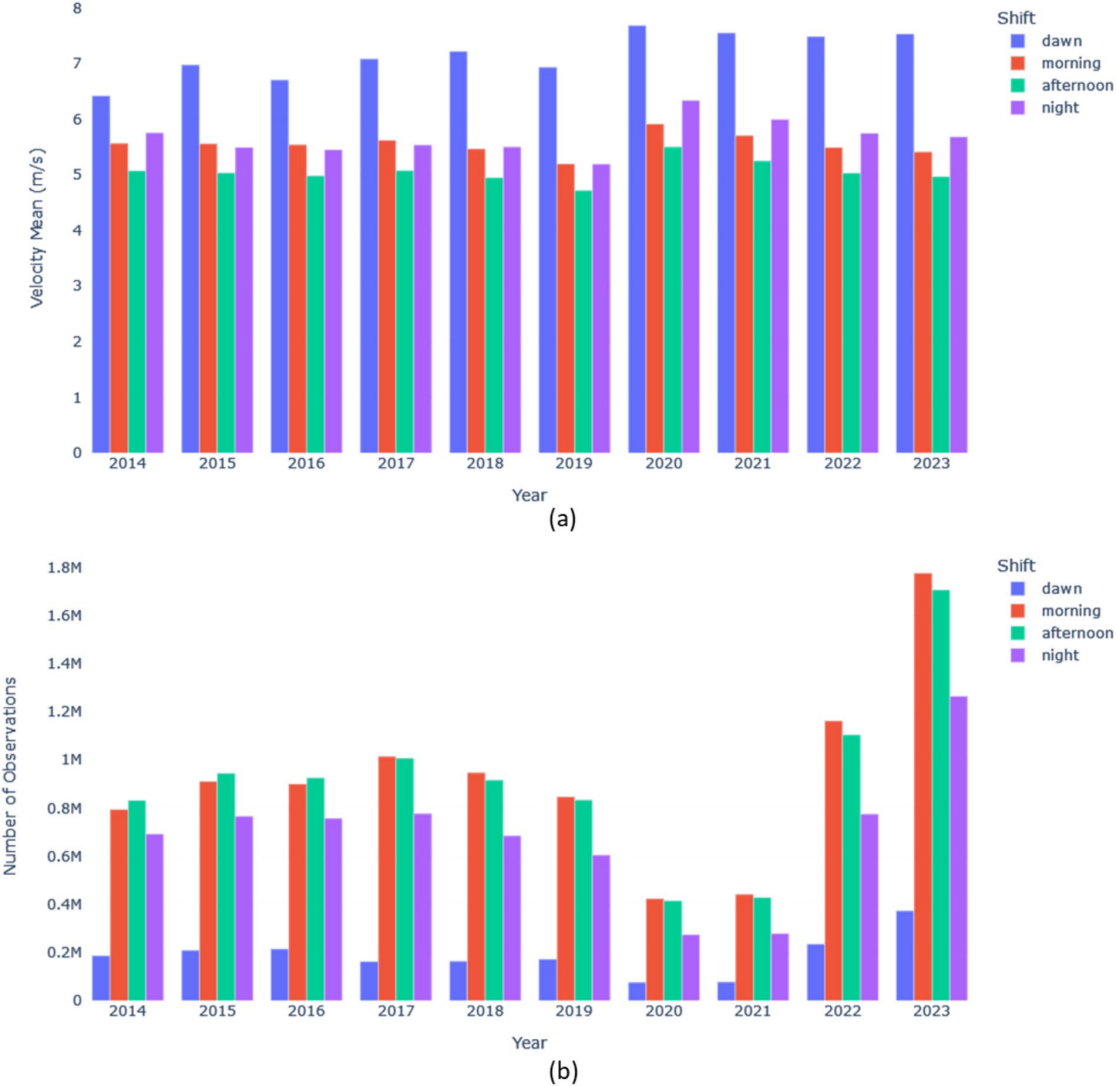
On each figure, the horizontal axis shows the year. The vertical axis shows (**a**) the mean of the instant velocity (measured by the GPS instrument embedded into the vehicle) on each shift per vehicle in the database and (**b**) the average number of observations per shift in the database.

We specifically focus on observations of buses in circulation, excluding those indicating buses stopped in parking areas or terminals. Table 7 Notably, the night and morning shifts exhibit the highest average speeds, which aligns with expectations, given the reduced street activity during these periods. Figure 7(a) illustrates the average bus speed per shift (dawn, morning, afternoon, and night) for each year, segmented based on Table 8. Night and morning shifts exhibit the highest average speeds, reflecting reduced street activity during those periods, as further evidenced in Fig. 7(b), which portrays the average number of observations for each shift across the years.

**Table 7:** Rainfall Zones.

Rainfall Zone ID	Rainfall Zone Names
1	Vidigal
2	Urca
3	Rocinha
4	Tijuca
5	Santa Teresa
6	Copacabana
7	Grajaú
8	Ilha do Governador
9	Penha
10	Madureira
11	Irajá
12	Bangu
13	Piedade
14	Jacarepaguá/Tanque
15	Saúde
16	Jardim Botânico
17	Barra/Itanhangá
18	Jacarepaguá/Cidade de Deus
19	Barra/Rio Centro
20	Guaratiba
21	Estrada Grajaú/Jacarepaguá
22	Santa Cruz
23	Grande Méier
24	Anchieta
25	Grota Funda
26	Campo Grande
27	Sepetiba
28	Alto da Boa Vista
29	Av. Brasil/Mendanha
30	Recreio dos Bandeirantes
31	Laranjeiras
32	São Cristóvão
33	Tijuca/Muda

**Table 8:** The DST attributes correspond to the variable used.

Shift	Time
Dawn	00:00 am to 05:59 am
Morning	06:00 am to 11:59 am
Afternoon	00:00 pm to 05:59 pm
Night	06:00 pm to 11:59 pm

**Fig. 8:**
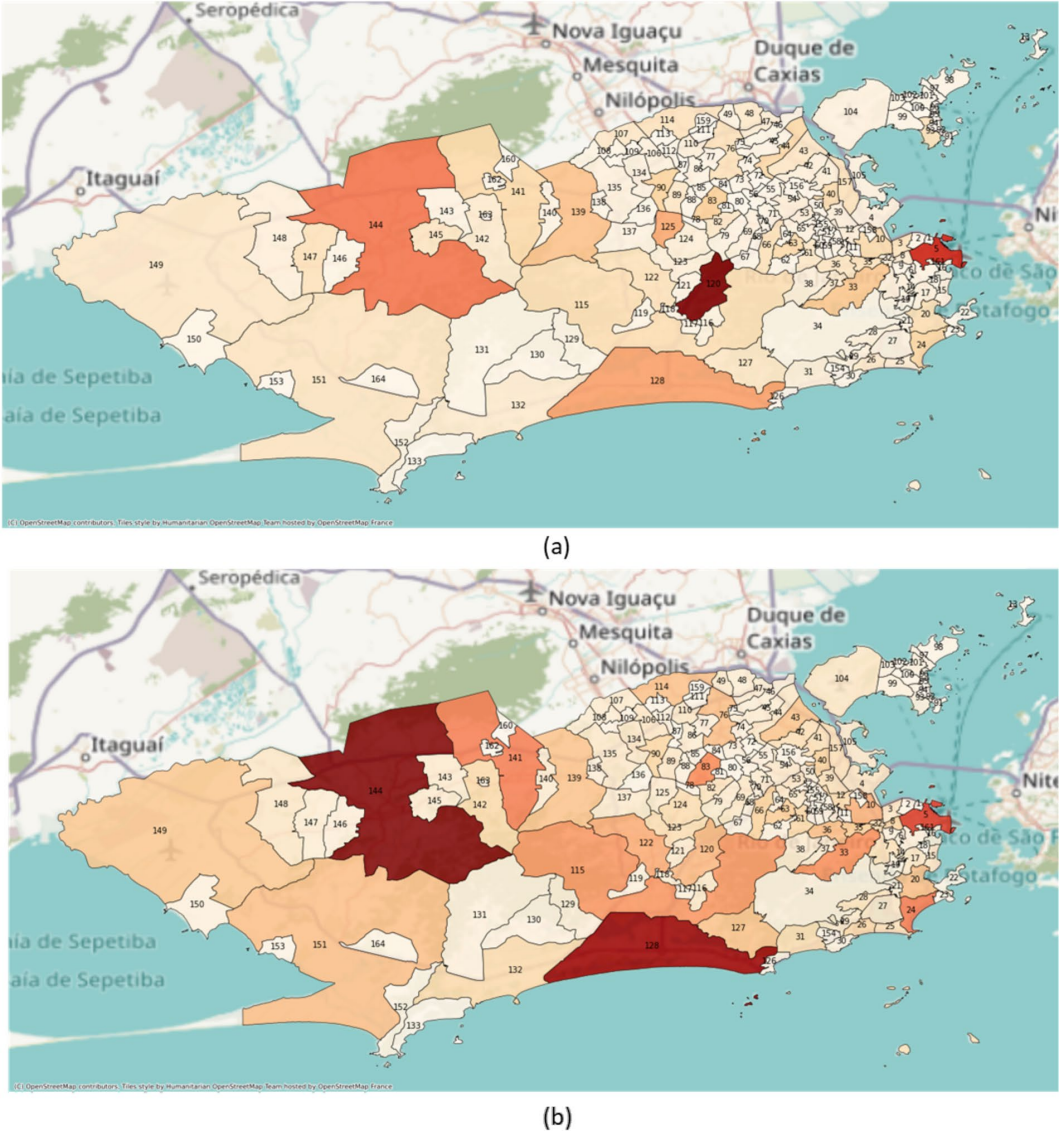
Neighborhoods in Rio de Janeiro: (**a**) shows the number of observations per neighborhood, and (**b**) depicts the mass of CO gas emitted by vehicles from 2013 to 2023. Lower values are associated with lighter colors and higher values with darker ones. Table 2 provides the correspondence between ID numbers and Neighborhoods.

Figure 8(a) presents a heat map depicting the total number of observations by neighborhood. Some neighborhoods, such as Freguesia/Jacarepaguá (ID:120), Campo Grande (ID:144), and Centro (ID:5), stand out with more circulating buses. In Fig. 8(b), the heat map represents the mass of CO emitted in each neighborhood throughout the evaluation period, indicating potentially poorer air quality in smaller areas regardless of air circulation considerations (more detail in^15^).

## Usage Notes

The Carioca_MapBus dataset provides historical data on urban mobility in Rio de Janeiro. It can enrich various research projects, such as analyzing urban mobility in specific periods or regions or broader studies that incorporate additional information from DSTs. For example, researchers can explore correlations between urban mobility and environmental factors such as rainfall and air pollution. Below, we provide more examples drawn from our research.

One potential research topic involves analyzing bus density during different time slots and in specific city regions. Increased density may cause traffic jams, even if it does not significantly impact average speed. If we find this correlation, we can explore creating specific interval routes or dedicated bus traffic lanes to enhance average speeds in the region.

Rainfall information is also available in the dataset, enabling researchers to evaluate the speed of buses in times of rain and helping to identify rainfall zones that suffer significant consequences for urban mobility due to rain. Public authorities can then direct efforts to these locations and anticipate risks based on rain forecasts.

The dataset also allows for the assimilation of rainfall data with bus observations. This way, researchers can obtain the volume of rain the bus experienced during its route, providing the identification of possible flooding points.

**Fig. 9:**
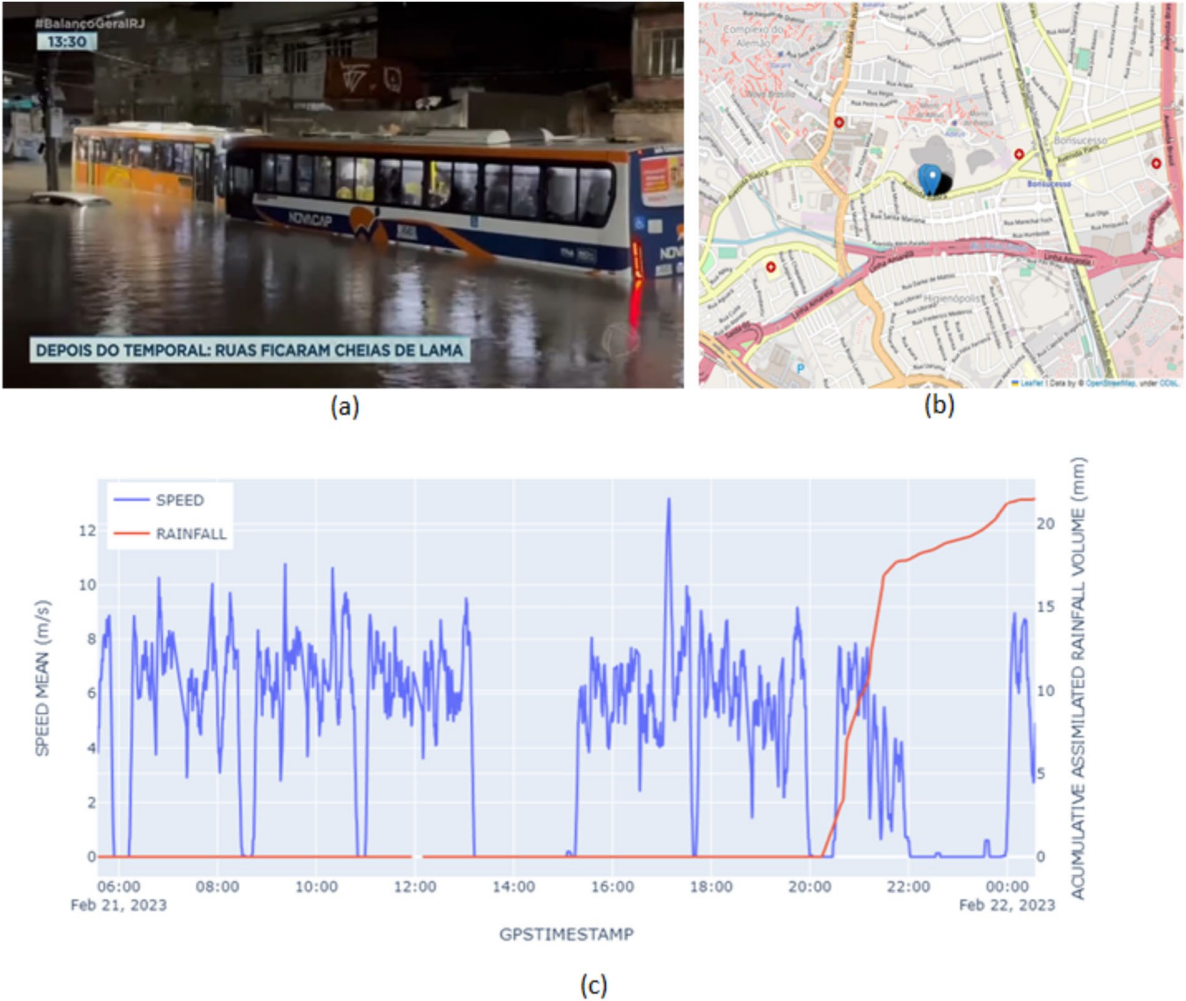
Buses stopped in a flooded area in the Bonsucesso neighborhood. Source: TV news broadcast from RecordTV (**a**), and the geo-position information in the database recorded for B51518 bus between 10:00 pm and 11:30 pm (**b**). The moving average speed in the last 5 minutes (blue) of the B51518 vehicle and the accumulated rainfall (red) experienced by it on February 22, 2023 (**c**).

To exemplify this scenario, we identified the occurrence of heavy rains on February 22, 2023, in the city of Rio de Janeiro, based on a TV news broadcast from RecordTV^9^. In the news, Fig. 9(a) presents two buses stopped in a flooded area in the Bonsucesso neighborhood. From the search for this information in the Carioca_MapBus dataset, we identified that one of the buses stopped in the flood refers to the bus on line 917, whose identification is B51518. Figure 9(c) shows the moving average speed in the last 5 minutes of this bus and the accumulated rainfall experienced by it on February 22, 2023. Note that from 8 pm onwards, the volume of rain increases and that between 10 pm and 0 am, the bus remains practically at a standstill. Figure 9(b) illustrates the region with the stopped vehicle. As reported in the news, the location is Avenida Itaoca in the Bonsucesso neighborhood. This analysis highlights the possibility of using the Carioca_MapBus dataset to identify possible flooding points.

Another potential research topic is using the information on pollutant gas emissions to correlate this data with bus movements and assisting the government in proposing measures to mitigate emissions from the city’s public transportation, such as reducing routes, changing trajectories, or decreasing the number of buses on specific routes. Geotagging and estimated emissions are the building blocks for spatiotemporal and traffic congestion analysis of pollutant emissions.

It is important to note that although buses are the predominant mode of transport in Rio de Janeiro’s urban mobility, they are not the only public transport in the city. However, as seen in Fig. 3(a), the bus lines have an operating network covering the entire city, which supports understanding the city’s urban transport quality.

Overall, the availability of the Carioca_MapBus dataset significantly advances studying urban mobility in Rio de Janeiro, combining data from bus observation with positional information on neighborhoods and rainfall regions, rainfall volumes, and pollutant gas emissions. By providing the dataset construction process, it becomes possible to reproduce the study with new data to observe new insights and improvements.

## Data Availability

The pipeline that generates the data is published on GitHub and is available^9^. The query code corresponds to “dst-retrieval.py” file and we also provide a PDF document (Readme) that describes its usage and is availabe on https://osf.io/6h3wy.
